# Applying a complex adaptive system's understanding of health to primary care

**DOI:** 10.12688/f1000research.9042.2

**Published:** 2016-09-16

**Authors:** Johannes Bircher, Eckhart G. Hahn

**Affiliations:** 1 Department of Hepatology, University of Bern, Meikirch, CH-3045, Switzerland; 2 Department of Medicine 1, University Hospital Erlangen, Erlangen, D-91054, Germany

**Keywords:** Meikirch model, health, complex adaptive system, primary care, internal medicine, family medicine, diagnosis, therapy

## Abstract

This paper explores the diagnostic and therapeutic potential of a new concept of health. Investigations into the nature of health have led to a new definition that explains health as a complex adaptive system (CAS) and is based on five components (a-e). Humans like all biological creatures must satisfactorily respond to (a) the demands of life. For this purpose they need (b) a biologically given potential (BGP) and (c) a personally acquired potential (PAP). These properties of individuals are embedded within (d) social and (e) environmental determinants of health. Between these five components of health there are 10 complex interactions that justify viewing health as a CAS. In each patient, the current state of health as a CAS evolved from the past, will move forward to a new future, and has to be analyzed and treated as an autonomous whole. A diagnostic procedure is suggested as follows: together with the patient, the five components and 10 complex interactions are assessed. This may help patients to better understand their situations and to recognize possible next steps that may be useful in order to evolve toward better health by themselves. In this process mutual trust in the patient-physician interaction is critical. The described approach offers new possibilities for helping patients improve their health prospects.

## Introduction

Individuals consult their physicians when they feel something is out of order, e.g. when they experience pain, fatigue or some other disorder. Physicians then examine them and specifically look for pathological changes. After such an investigation they make a provisional diagnosis and explore their patients further or treat them according to what has been discovered. This approach and type of thinking goes back to the pathologist Rudolf Virchow who, in 1858, used 20 lectures to describe “cellular pathology”, a characterization of different diseases ^1^. Although the foundations of medicine have evolved since then, the general principles of medical practice have remained the same. Only over the past 20 years has complexity science gradually entered into medicine ^2, 3^. This has become particularly important for the interpretation of health and disease as different states of a complex adaptive system (CAS). The Meikirch model is a new definition of health that exhibits all the features of a CAS ^4^. For such systems the concepts based on Virchow’s pathology ideas are no longer adaquate. An understanding of health and disease now requires appreciation of complexity science which introduces a new dimension for diagnosing and treating patients. It includes the potential of improving health in a way that was hitherto practiced only exceptionally. The purpose of this paper is to summarize the relevant features of the Meikirch model and to spell out in detail how the model and complexity science may be applied for a better understanding of a patient’s disease and its treatment.

## The Meikirch model: definition of health and disease

**Figure 1.**
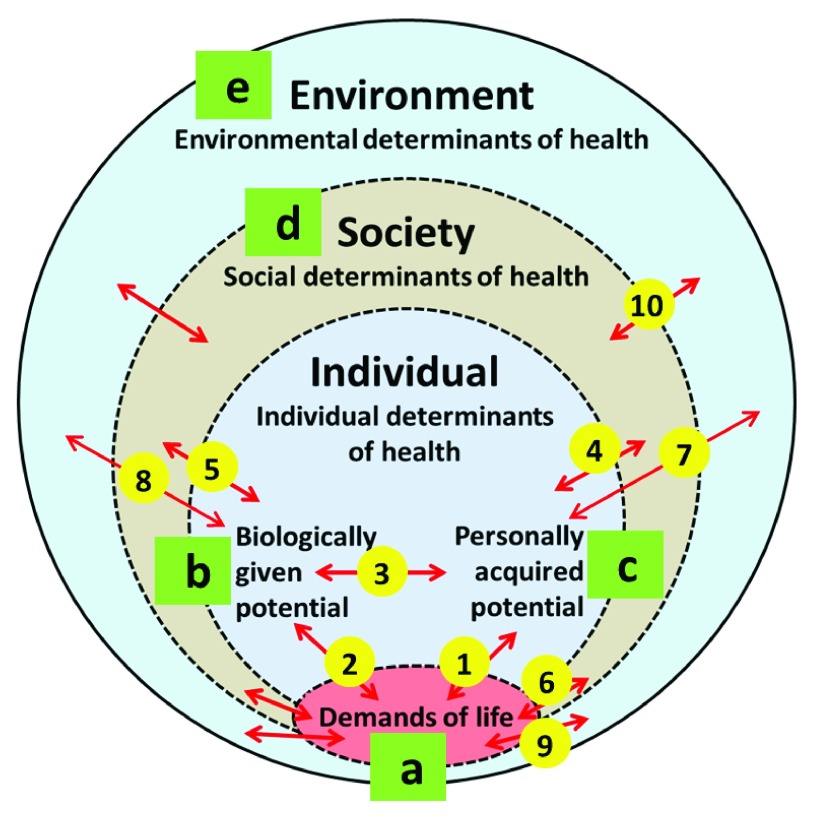
The Meikirch model consists of five components (a–e) and ten complex interactions (1–10).

The Meikirch model is based on five components ( Box 1) and 10 complex interactions ( Figure 1). This framework allows us to define health and disease as a complex adaptive system ( Box 2). Figure 1 depicts the five components from *a* to *e*. The interactions are exhibited as double-edged arrows from *1 – 10*. A short explanation of the five components and their interactions is presented below. The complete description of the model with its scientific background is given in the previous publications ^4, 5^.

Box 1. **Meikirch model: The five interacting components of health ^4, 5^**

**a.**  Life's demands
**b.**  Biologically given potential (BGP)
**c.**  Personally acquired potential (PAP)
**d.**  Social determinants
**e.**  Environmental determinants

Box 2. **Wording of the Meikirch model i.e. the definition of health and disease ^4, 5^**

**1.**  Health is a dynamic state of wellbeing emergent from conducive interactions between an individual's potentials, life's demands, and social and environmental determinants.
**2.**  Health results throughout the life course when an individual's potentials and social and environmental determinants suffice to respond satisfactorily to the demands of life. Life's demands can be physiological, psychosocial, and environmental, and vary across individuals and contexts, but in every case unsatisfactory responses lead to disease.

Each human being must fullfil the demands of his or her ***life situation*** ^6^. Physiological, psychosocial and environmental demands vary with time and circumstances. *Physiological demands* are related to the homeokinetic balance of nutrients, energy and water necessary for maintaining bodily functions including procreation (examples are work, pregnancy, childbirth and brain function). *Psychosocial demands* are the individual´s exposure and response to social conditions needed to succeed in social integration and mental, personal and spiritual development. Expectations and roles related to work, family and society as a whole combine with personal aspirations, values and lifestyle in changing settings and contexts. This also includes peace with the fact that every human being must die. *Environmental demands* include the availability of and immediate or latent threats from living conditions (e.g., water, nutrients, climate, radioactivity, pollutants, carcinogens, workplace conditions).

The potential of an individual to meet the demands of life is partly biological e.g. a gift of nature - ***biologically given potential (BGP)***, and partly acquired during life - ***personally acquired potential (PAP).*** At birth the BGP is based on the genetic equipment, epigenetic regulation and quality of the pregnancy. The BGP diminishes throughout life and is zero at the time of death. During the lifetime the BGP may be threatened or damaged by socioeconomic disadvantages, diseases, injuries and/or defects. The PAP results from the entirety of physiological, mental, spiritual and social resources acquired during the lifetime. It may continue to grow when a person cares for it. Social and environmental conditions also influence the growth of the PAP by providing or withholding determinants of health.

***Social determinants of health*** strongly interact with the demands of life and the potentials of the individual ^4^. Equity and equality, social concerns, working conditions, autonomy and social participation affect health and longevity ^7, 8^ and are also major determinants. Likewise, ***environmental determinants of health*** are factors in living and working conditions that affect each person. They may sometimes be of global significance like natural resources, catastrophes, population growth and climate change ^9, 10^.

Based on these five components and their interactions with each other the Meikirch model represents a new definition of health and disease as shown in Box 2. Possible individual and public health care outcomes as a result of a hypothetical implementation of the Meikirch model have been discussed elsewhere and suggestions for clinical and health systems research have been made ^5^.

## Health as a Complex Adaptive System (CAS)

**Figure 2.**
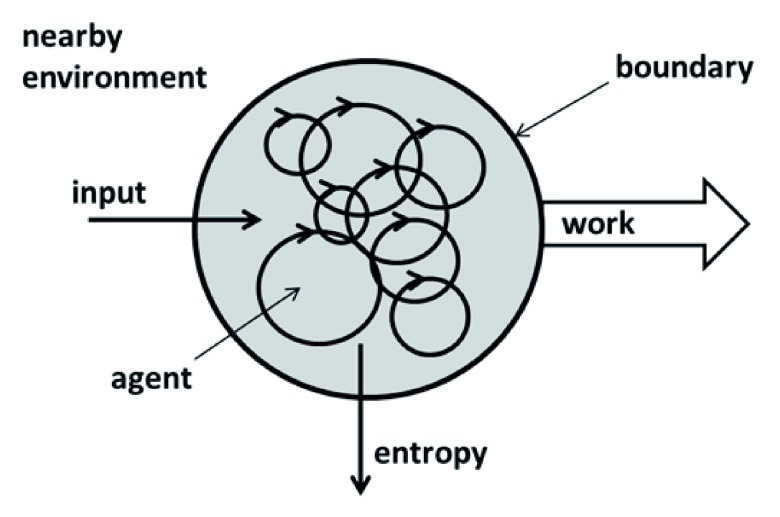
General model of a complex adaptive system (CAS).

A complex adaptive system is an entity with a more or less permeable boundary between it and its nearby environment ( Figure 2) ^11^. It can take up material and energy from the environment (input), release end products (output, e.g. entropy) and do work. Within the system there are many different parts called agents. In Figure 2 they are symbolized as circles. They continuously and autonomously interact with each other in a nonlinear manner, contributing to the product, the so-called emergence of the CAS. The term emergence indicates a new and often unpredictable quality which is more than the sum of the functions of each part. A CAS always functions as a whole. A CAS is equipped with a learning and bonus arrangement for the interactions among its agents. This gives it the possibility to adapt to changes in the environment, i.e. to learn. If for some reason this adaptation functions poorly, the CAS suffers. If it does not function at all the CAS becomes chaotic, goes into a crisis or vanishes. Repeated minor critical disturbances may lead to the so-called butterfly effect ^2^. This effect refers to the image of a butterfly that flaps its wings in South America and induces a hurricane in Texas. Examples of medical conditions where a similar mechanism may lead from minor incidents to major consequences are ventricular fibrillation, epileptic seizures, tantrum, and psychotic states. Every CAS has evolved from a prior condition and autonomously progresses toward an unforeseeable future state. A CAS may be part of a larger CAS or be composed of many CASs. Such structures are called nested CASs.

In the Meikirch model the five components (a–e) including their subcomponents are regarded as agents ( Figure 1). Interacting with each other they spontaneously arrange themselves in such a way that the evolving emergence, i.e. the state of health, is the result of the functioning of the system as a whole. In each case a specific working-arrangement is in operation, but it is not necessarily the best solution for the system. Energy flow in humans has been called vitality, drive, or sense of purpose. This indicates that energy flow may be material and immaterial, e.g. based on a desire to be loved, on a pursuit of values, or on living for a spiritual purpose. Investigation of the material and immaterial double nature of human energy flow may help toward a better understanding of a person´s health.

An entire life is an evolutionary process. Biologically human life originates with the fertilization of an oocyte. This then passes through the stages of embryo and foetus to the maturity needed for birth. At some time during intrauterine life the personality of the individual is created. At least, physicians and midwifes say that in a new-born it is clearly recognizable. From then on the complete Meikirch model is fully operational throughout all the life course phases of each person. Thus evolution from birth to death demonstrates clearly how many adaptive processes occur as part of the different complex interactions described by the model. While the two potentials evolve, the demands of life, the social, and the environmental determinants also vary. Within these limiting and supporting contexts individuals follow an autonomously chosen course of life. Under such conditions it is not surprizing that some adaptations may not be fully successful for some period of time or permanently. Challenging examples are malnutrition, infectious diseases, love deprivation, sexual maturation, pregnancy, genetic defects, professional stress, the raising of children, physical involution, aging, etc. Such changes may lead the health of an individual as a system into a state of crisis. If it is minor, the two potentials may still manage the demands of life and the difficulties may resolve spontaneously after some time. Such situations may represent illnesses, but are not considered as disease or pathological. They may, though, evolve into a chronic state that draws energy from the person and thereby may explain, for instance, insomnia, chronic fatigue, or somatoform symptoms. If the defect becomes more severe it may lead to a disease that requires more medical attention. In the Meikirch model the term “disease” implies that for some reason one or several adaptation processes are not successful enough to empower the two potentials to satisfy the demands of life.

## Methods

To allow for future research, hypothetical consequences of the Meikirch model and of the properties of CASs have been explored with the purpose of better understanding the state of health of patients, particularly in primary care. According to the Meikirch model, health of a healthy individual or of a patient is considered to be a nested CAS, composed of grouped CASs and being embedded in higher CASs. For the analysis of various conditions, the significance of each of the five components and of each interaction within the Meikirch model were visualized. In addition, possibilities of supporting favourable evolutions of the respective CAS and its meaning for the whole person (nested CAS) were analysed. In this process, the deduction (from observation to theory) and induction (from theory to observation) cycles were repeated until coherent results were achieved.

## Results

### Categories of illness and disease

Disturbances in health and healing follow a pattern that can be described by four categories:

**1.**  Minor maladaptations lead either immediately or with varying delays to discomfort (illness) or to signs of a disease. Examples for the former are minor acute infections or tension headaches, and for the latter, overweight, type 2 diabetes, or arterial hypertension.
**2.**  A more severe disturbance of the system leads it into a crisis, i.e. it becomes “chaotic”. Such states may be corrected spontaneously, for example, or by behavioural changes, or by interacting with a physician or healer, by medications, or by medical or surgical interventions. Thereafter there may not be an immediate complete recovery to health. The full adaptive evolution may take time and further interventions, i.e., convalescence or rehabilitation, may be needed. These phenomena may lead to complete healing or to healing with remaining defects.
**3.**  If a CAS is disturbed continuously for a prolonged time, the CAS may not be able to satisfactorily respond to the demands of life. This represents a chronic disease or invalidity. If the condition is progressive and serious, it may lead to death. Examples are rheumatic or degenerative diseases and different types of neoplasms.
**4.**  Considering a disturbed state of health as a maladapted CAS implies that patients cannot simply be healed by the actions of a competent physician. Healing is much rather the result of a process of self-reorganization, enabling the two potentials to again satisfactorily fulfil the demands of life. The task of physicians and other health professionals therefore consists in being competent advisors and fellow human beings that assist the patient in realizing the necessary evolution himself.

### Assessment of a patient´s health

It always is appropriate to examine patients with an ordinary medical history and physical examination to which all indicated laboratory tests and imaging procedures are added. Simultaneously it may be purposeful to perform an analysis of the patients´ health as a CAS. In this case the five components and the ten complex interactions of the Meikirch model are assessed by an extended history as exemplified in Box 3. This process assures that all possibly important aspects are evaluated. A thorough analysis will give patients a new way to look at their health and how they had conducted their lives. They will discover aspects they did not think about before, and this may be of therapeutic value. At the same time the physician may start to interpret the patients´ history and findings in a new way. This may discover further possibilities for helping a patient to autonomously evolve to a new state which hopefully comes closer to health.

Box 3. **Assessment of a patient’s health by taking a history focussing on all aspects of the Meikirch model**

In addition to the five components and the ten complex interactions the energy flow and other patterns of the model also need to be investigated. The questions enumerated are just examples that have to be adapted and complemented further as needed for each patient’s specific problems.

**Questions related to the components of the model (a–e):**

**a)**  What specifically are the demands of life to which the patient has to respond?
**b)**  How does the patient perceive the evolution of his or her physical health?
**c)**  How does the patient feel about him- or herself? Can he or she manage themselves? Does he or she invest in the future?
**d)**  How is the patient integrated into family, household, friends, society and government?
**e)**  In which type of natural environment does the patient live?

**Questions about the interactions (1–10)**

**1.**    How does the physical body of the patient (past and current) interact with the demands of life?
**2.**    How does the patient deal with his or her physical, psychological and spiritual demands of life?
**3.**    How does the patient interact with him- or herself, especially with the body? Does he or she invest in it?
**4.**    How does the patient interact with family, household, friends, and government etc.?
**5.**    How does the physical body of the patient interact with the society (past, present, future)?
**6.**    How does the society influence the demands of life?
**7.**    What tis the attitude of the patient toward his natural environment?
**8.**    Which are the past, present and future interactions of the natural environment with the patient’s physical body?
**9.**    How does the natural environment modify the patient’s demands of life?
**10.**   How does the society interact with his or her natural environment?

**Questions about vitality, motivation and purpose in life**

- What is the source of the patient’s vitality? Is it spontaneous or rather focused on objectives or purposes? Which occasions induce which type of vigour? What is his or her purpose in life?
- How is the patient’s physical, intellectual, and emotional vitality? How much is hedonistic and how much eudaimonic?
- What does the patient do with his or her vitality? Is it used mostly in family, profession, or hobbies?
- What is the energy flow between the patient and the physician like?

**Questions about temporal patterns**

- When did the patient feel completely healthy the last time? When and how did he or she loose health or wellbeing?
- What were the manifestations of the crisis?
- How was the time course of the disease up to now? Which factors induced aggravation and which brought about improvements? Which changes within or outside the patient induced which type of changes?
- What is the explanation of the patient for the current state of health and for the failure to improve it? What does the patient need in order to surmount the present crisis?
- What are the future plans of the patient? How much sense of purpose do they evoke?

### Treatment

Obviously, for all medically diagnosed conditions treatments are to be prescribed as indicated. Yet, in medicine, indications generally leave considerable room for judgements. Thus the findings collected by assessing all the components and interactions of the Meikirch model must be considered and integrated as far as possible. A CAS cannot be manipulated to health. It must be assisted as it reorganizes itself autonomously to a new state, in order to better fulfil the demands of life and hence better health and well-being. The role of the physician, therefore, is to accompany the patient during the process he goes through. Some advice, assistance, or therapeutic intervention may be helpful, but only the patients are in a position to create their new future state for themselves. By analysing their condition as a CAS together with their physician including all components and all interactions of the model they presumably receive many new ideas that they can use to emerge into a healthier state in the future. For example, they may want to make up their mind whether or not they will accept all the conditions that have determined their life in the past. In this respect, a discussion with their physician of alternatives with their consequences may be useful. For many patients it might be constructive to deal with the energy flow in their system, e.g. to speak about the purpose of their lives.

The process of reorientation based on the Meikirch model takes time. During this period it may help the patient if they find in their pysician a trustworthy human beeing with whom they can discuss all sorts of alternatives. Ultimately though, patients have to create their own future. It will encourage them if they feel understood, trusted and accompanied by an experienced person with a sincere interest in their wellbeing.

### When is attention to the Meikirch model and the principles of a CAS indicated?

In primary care, such as general internal medicine and general practice, there are many patients who come for consultations because they feel ill. Yet, on examination no clear pathology is found. Thus far such complaints have been explained as functional and often were regarded by physicians as unimportant. Patients then received drugs that may be symptomatically beneficial or placebos or, more often than not, harmful or nocebos. Instead of acting with benign neglect, the Meikirch model offers a true and positive alternative approach. In many cases it will help the patients to understand their problems, to readjust their potentials and to advance their readaptation to the demands of life. Thereby patients may again come closer to a state of health and wellbeing.

## Discussion

At the present time the Meikirch model is a hypothesis grounded on a theoretical and conceptual framework. Yet, up till now much of health care has not been concerned with an understanding of the *nature* of health; it used instead an intuitive notion of wellbeing which did not lead to new insights. In contrast, a rational understanding of health - as given by the Meikirch model - offers innovative opportunities. Today this model is better founded on scientific evidence than other definitions of health. Its ultimate validity, however, will be documented only by using and evaluating it in practice. This must be done with due consideration to the special features of the model. Much further research is urgently needed.

For the past 150 years medicine has been working with methods derived from Newtonian natural science and obviously has achieved major advances. They are based, however, predominantly on materialism and neglect the social and spiritual features of human nature. In addition, until recently medicine has not considered systems theory. It appears that these two aspects offer new opportunities for health care to become even more effective. Systems thinking implies that science based on Newton must be complemented by complexity science. Particularly for the purpose of health care a phenomenological, narrative, and evolutionary holism must be added to analytical reductionism ^11^. Poorly functioning parts are not simply corrected by appropriate drugs or surgical operations. Instead considerations of the evolution of the patient’s health to its present state, earlier successes in self-management and failures in the handling of his present crises can be evaluated. Here, Antonovsky’s sense of coherence and meaningfulness also may be very helpful ^12^. The necessary changes a patient has to realize must not come top-down from the physician, but rather bottom-up, originating in the patients themselves, e.g., via new insights. For this purpose mutually trusting patient-physician interactions are critical for a successful future: the physician must believe in the patient’s abilities to evolve to a new state and must accompany and support him with loving wisdom in this endeavour.

When speaking with older and experienced general practitioners, and when reading about how they managed their difficult patients, it becomes evident, that they knew their patients from the past quite well. In many difficult situations they often had to accompany rather than to treat them. Such patients remained very loyal because they understood what their doctor had contributed to their health. At the same time physicians realized that they had nothing more to offer than their personal support as a professional human being. The Meikirch model offers now a rational approach to such difficult cases and it is hoped that the five components and the ten complex interactions will lead to new opportunities for patients to move toward better health. At the first glance the described system’s approach to patient care is similar to what Michael Balint intended with his groups ^13^. He was a psychiatrist and pursued the purpose of “Training General Practitioners in Psychotherapy” to better understand and respond to the needs of their difficult patients. In contrast, the systems theory approach focusses on a new look at a patient’s possible unresolved evolutionary steps, analyses the biological given and personally acquired potentials and offers him an opportunity to progress further in his personal biography. More research is needed to validate the promises and limitations of this methodology.

The Meikirch model distinguishes two types of very different potentials with which the demands of life must be met, the biologically given potential (BGP) and the personally acquired potential (PAP). The latter is the resource that continuously pilots the adjustment to new life situations. It is the locus of executive functions ^14^. For this purpose it interacts with all components of the system. The PAP is the seat of memory, visions, fantasy, reasoning, attentional control and inhibitory control, and problem solving. Its sustained evolution toward more and more wisdom is critical for the maintenance of health. The PAP can learn to compensate in part for losses of the BGP. This leads to an interesting aspect of the relationship between the two potentials. It may be compared to rider and horse. If the rider wants that his horse serves him well he has to take good care of his horse. It appears that the neglect of the PAP in modern medicine is well perceived by patients and they have turned to complementary or alternative medicine. In fact, much of the success of homeopathy and other methods might be explained by the physician/patient interaction with its effects on the complex adaptive system that expresses the patient’s health. This mechanism may be relevant also for much of the success of other complementary or alternative treatments. It is our opinion, however, that it will be better to work with the CAS in a planned and scientifically justifiable manner based on the Meikirch model than to apply unproven methods. The model would also serve as an excellent framework for a proper practice of evidence based medicine as defined by David L. Sackett: “Evidence-based medicine (EBM) requires the integration of the best research evidence with our clinical expertise and our patient’s unique values and circumstances ^15^.” This applies also to the newly evolving holistic clinical approach “Integrative Medicine and Health” that “reaffirms the importance of the relationship between practitioner and patient, focuses on the whole person, is informed by evidence, and makes use of all appropriate therapeutic and lifestyle approaches, healthcare professionals and disciplines to achieve optimal health” ^16^.

When looking at health as a lifelong and complex evolutionary process, it is not surprising that crises do occur frequently. Throughout human life there are several major and many minor evolutionary steps to be taken. End of breastfeeding, beginning of school, puberty, professional formation and advancement, partnership, family, menopause, and involution of old age are some of the more demanding processes. Today they must be overcome in a society that offers insufficient respect for the personality of each individual. Lack of a motivating purpose in life and insufficient social support have become almost normal. Economic exploitation, power plays, isolation, social neglect and even wars weigh heavy on the demands of life. A culture that is really concerned with the health and wellbeing of its individuals needs to strongly support lifelong human development by investing in life-affirming compassion and truth ^5^. The Meikirch model provides a framework for how this could be achieved.
