# Supplementary material for: Applying a complex adaptive system's understanding of health to primary care
Source: F1000Res. 2016 Sep 16;5:1672. Originally published 2016 Jul 12. [Version 2] doi: 10.12688/f1000research.9042.2 (PMC5043445; doi:10.12688/f1000research.9042.2)
Supplement: Peer review history

# Peer review: Referee response for version 2

**DOI:** 10.5256/f1000research.10370.r16661

**Published:** 2016-09-28

**Authors:** Dianne Delva

**Affiliations:** 1 Department of Family Medicine, Faculty of Medicine, University of Ottawa, Ottawa, ON, Canada

I would like to thank the authors for their explanations and revisions of the paper: Applying a complex adaptive system's understanding of health to primary care.

 It is very clear, provides much food for thought in thinking about health and disease in people as complex adaptive systems. The paper provides a framework for considering the variability in how patients adapt to various conditions. It also centers the responses on the patient and their “Healing is much rather the result of a process of self-reorganization, enabling the two potentials to again satisfactorily fulfil the demands of life.” And helps the physician to know that compassion, humanism, creativity and willingness to stand by are legitimate roles.

I have read this submission. I believe that I have an appropriate level of expertise to confirm that it is of an acceptable scientific standard.

# Peer review: Referee response for version 1

**DOI:** 10.5256/f1000research.9730.r15463

**Published:** 2016-08-05

**Authors:** Dianne Delva

**Affiliations:** 1 Department of Family Medicine, Faculty of Medicine, University of Ottawa, Ottawa, ON, Canada

This is an article describing the confluence of two theories, the model of health as defined by the authors in the Meikirch model which appears to be a model developed and published by the authors, and complex adaptive systems.  The authors propose this as a method to better help patients adapt and aim towards improved health. 

**Strengths:**

 The authors have developed a theory and are proposing this explicit theory can better support patient care.  Primary care is known for its complexity and uncertainty. Thus the system is a good framework for thinking about a holistic approach to patient care and to the variability of adaptation based on the components. The categories of illness and disease would be helpful for learners to understand that not all illness has a desired resolution. The comments on complex adaptive systems as a way of understanding health in individuals and society is useful in reminding us that linear models often ignore the unintended consequences of change. 

**Suggestions:**

 As a family physician, I found the paper both interesting and difficult to follow.  In general the language is clear with minor errors.  I am not an expert on complex adaptive systems but agree with the comment regarding the use of some terms as detracting from the premise of the article. 

 My main suggestion would be to expand the methods section.  It is not clear to me who was involved in the process, how this was carried out.  I wonder if this would be better as a theoretical proposal rather than a scientific study.  The examples chosen seem to be the opinions of the authors and have not been tested.  Some are quite vague and would require fuller explanation. 

 I also agree that comparing this theory to others might be useful.  McWhinney is referenced and as a leader in Family Medicine in Canada, the four principles of family medicine and a model we use to teach residents for patient-centered care helps us to provide care in family medicine that is not likely to be enhanced by the model.  The four principles are:

  

- The family physician is a skilled clinician.
- Family medicine is a community-based discipline.
- The family physician is a resource to a defined practice population.
- The patient-physician relationship is central to the role of the family physician.

It may be that the authors are specialists and thus are less familiar with general/family practice. The idea that “Physicians then examine them and specifically look for pathological changes.” is not the premise for family medicine in Canada.  The four principles address the environment and social determinants of health in situating the patient in the community and family in which they live.  The idea of accompanying the patient resides in the value of continuity of care and the patient-physician relationship. Knowing the patient’s values and past helps to tailor the approach to support the patient in the direction they wish to go. The skilled clinician is a critical thinker who can deal with uncertainty and as a resource to the practice population advocates for the system to better serve the population. 

 In Canada we teach a model of interviewing that includes the traditional history focused on disease interwoven with the IFFE model. These include the patient’s ideas of what is wrong with them, their fears, how their function is affected and their expectations from the physician/system. These questions are essential to the patient-centered approach and situate the consultation clearly with the patient as the CAS. 

 In particular, I find the suggestions for taking a history based on the model cumbersome and clearly the language would be challenging for patients. It is also not clear to me how these questions are related to the CAS. 

 The treatment approach mirrors the patient-centered approach taken in family medicine. We know that patients often do not fill prescriptions and asking patients what might work for them or what they are comfortable with, helps one to support a treatment approach that is right for the particular patient.  Being aware that a patient does not have the resources to access a particular treatment is essential to a collaborative effort for care.

 Some of the examples either require more explanation or less linear thinking. The idea that *in utero* does not include the environment overlooks the social determinants of health of the mother and their effect on the *in utero* environment. 

 I have been involved in a number of Balint groups and recall reading Michael Balint’s book. The statement “He was psychiatrist and pursued the purpose to train general practitioners in psychotherapy.” does not reflect my experience. My understanding was he focused on Difficult Clinician-patient relationships to help general practitioners interact and “accompany” our patients more effectively. The groups were meant to support physicians in their own context. I did not see this as training in psychotherapy. 

**Minor suggestions**

 “Examples of medical conditions are ventricular fibrillation, epileptic seizures, tantrum, or psychotic states.” This statement needs further explanation.  It is not logical from the discussion if we think of health in a holistic manner. 

 There may be cultural differences in writing and I appreciate English may not be the first language of the authors; however I find it a poor reflection to refer to all patients and physicians as male. 

I have read this submission. I believe that I have an appropriate level of expertise to confirm that it is of an acceptable scientific standard, however I have significant reservations, as outlined above.

# Response

**Published:** 2016-09-19

**Authors:** Eckhart G. Hahn

**Affiliations:** Univeristy Hospital Erlangen, Germany

**Response to the comments by Dr. Dianne Delva**

 We as authors appreciate the care Dr. Delva has devoted to our paper. It has helped us significantly to improve the manuscript and make it readable for family physicians. Also, we are happy that she recognizes the overall strengths of our contribution. We understand that she appreciated the value of thinking in terms of health and disease as complex adaptive systems (CAS) to embrace uncertainty and a holistic approach to patient care. In order to respond to her concerns with some aspects of our manuscript we would offer the following point by point response. We will also indicate where we have made changes and corrections of the manuscript.

1. From Dr. Delva´s critical comments as a family physician we have come to the conclusion that the original title does not completely reflect the content of the paper and may be misinterpreted. We do not intend to challenge any of the established models as applied to family medicine. We rather intended to explore the possible results of applying the principles of complex adaptive system´s thinking in primary patient care, possibly extending the present practice. Therefore, to avoid any misunderstanding, we have changed the title of the paper into “Applying a complex adaptive system´s understanding of health to primary care”. This was also suggested in the review of Dr. Sturmberg (accessible under http://f1000research.com/articles/5-1672/v1).
2. Interestingly, Dr. Delva refers to Dr. I. R. McWhinney, who had developed four principles for family medicine. He certainly is an important person for the principles of family medicine. These principles refer to the physician as a skilled clinician, to family medicine as a community based discipline, to family physicians as resource to a defined population, and view the patient-physician interaction as central. Interestingly these four postulates are also part of the Meikirch model. Also the proposals of the so called Wonca-tree are included in the model. The Meikirch Model is wider than the specific concepts for family medicine and therefore might in the future serve as an extended basis on which the others stand: this is what we suggest to explore.
3. We agree with Dr. Delva that the physician must value the continuity of care and be competent to deal with uncertainty. We, as proposed in the Meikirch model, have a different view how the patient should be supported, derived from the thinking of health and disease as a complex adaptive system. As a CAS, the patient´s situation evolves independently and autonomously, often against his or her will. The support in our opinion has the purpose to help the patient to move to his new state that results in a more satisfactory emergence. Often the route to this new state is not the “direction the patient wants to go”, but the direction to which the patient has to submit himself. That is why he needs the physicians support.
4. On page 4, an effort has been made to further clarify what a CAS means and what it may contribute to the primary care of patients. In figure 2 the term “attractor” was removed because it is not essential to understand the principle of a CAS.
5. On pages 5 and 6, the method section has been expanded as suggested by Dr. Delva. Both authors have contributed to the development and description of hypothetical consequences of the Meikirch model to stimulate future research. One of the authors (EGH) is trained in and practiced general internal medicine (and gastroenterology) and is familiar with Robert Nikolaus Braun´s conceptual frame of family medicine which is prevalent in German speaking countries.
6. In a participatory and integrative approach to the patient, the inclusion of the patient´s ideas, fears, functions and expectations (IFFE model) is indeed essential for any primary encounter between patient and physician. It is also part of the Meikirch model, as can be seen in box 3 on page 6. Dr. Delva finds it cumbersome to take a history based on the Meikirch model and states that it is not clear to her how the questions in box 3 are related to the CAS. Obviously this table contains examples, is not exclusive and must be adjusted to the patient’s specific problems. The purpose of box 3 is to give the physician a tool to make sure that the history he took covers the whole Meikirch model, i.e. is complete in this sense. The questions suggested in box 3, derived from the Meikirch model with its complex interactions, will also be the basis for describing the patient as a CAS. The Meikirch model is in itself based on complex adaptive systems thinking. Dr. Delva, is right in suggesting to use plain language in real life communication with patients.
7. In order to clarify the issue of the situation in utero we believe that the direct environment of a fetus is the uterus of the mother. The environmental and social determinants of the mother have nonetheless an indirect effect on the child´s development in utero as has been pointed out in a previous description of the Meikirch model.
8. Dr. Delva refers to her understanding of Balint groups. We have taken the information given in our paper directly from an original publication by Michael Balint (1896–1970) in the Br Med 1954 (see reference 13). Balint groups may have developed in a direction not intended by Balint himself, and we agree that the term psychotherapy is not well defined and can be understood in many ways in Balint´s original paper. The case reports included in this paper describe complex psychodynamic situations. We have changed our text on page 9 to stay as close to Balint´s original paper as possible.
9. The butterfly effect used as a metaphore on page 4 is now described in more detail to assure a better understanding of our mentioning of ventricular fibrillation, epileptic seizures, tantrum or psychotic states. These conditions, because of their dramatic appearance, are exclusively meant as an illustration of the butterfly effect.
10. Wherever the gender-neutral terms “patient” or “physician” were used in our text we have now avoided to use them in a gender-specific context by either omitting “he” or “she” or using the plural, including in the abstract.

Fink, W. & Kamenski, G. Wien Med Wochenschr (2009) 159: 173. doi:10.1007/s10354-009-0666-8

 Bircher J, Hahn EG: Understanding the nature of health: New perspectives for medicine and public health.

 Improved wellbeing at lower costs [version 1; referees: 2 approved]. *F1000Res.* 2016;5:167. 27134730

 10.12688/f1000research.7849.1 4837984. See page 4 „personally aquired potential“.

# Peer review: Referee response for version 1

**DOI:** 10.5256/f1000research.9730.r15187

**Published:** 2016-07-25

**Authors:** Joachim P Sturmberg

**Affiliations:** 1 Department of General Practice, University of Newcastle, Wamberal, NSW, Australia

This paper is an extension of the authors’ previous paper in this journal and stimulates long overdue debate. My previous comments ^1^ principally also apply to this paper.

 Two additional points:

  

1. The paper’s title “Health as a Complex Adaptive System: a new dimension of patient care in internal medicine and general practice” only loosely reflects the content of the paper, I would think that a title similar to “Applying a complex adaptive system’s understanding of health to clinical care” would be stronger and more appropriate. Offering clinicians alternative ways of practicing is certainly most welcome.
2. The paper - implicitly - wrestles to come to terms with the notions of health in relation to the “non-health” terms of illness and disease ^2^ . Even definable pathology is not objective as many studies have shown significant disagreement between experienced clinicians looking at the same “example of disease”. Highlighting the definitional problems and distinctions is especially important for primary care physicians as many people present with unspecific symptoms rather than a “specific diagnosis” ^3^. Diagnoses are socially constructed ^4^  and have become a “tyranny for clinicians” ^5^ . From a complexity perspective thinking about patients’ complaints as “conditions” or “states of being” clearly fosters more creative approaches to patient care and the emergence of more satisfactory outcomes.

I have read this submission. I believe that I have an appropriate level of expertise to confirm that it is of an acceptable scientific standard.

## References

[1] SturmbergJ: Referee Report For: Understanding the nature of health: New perspectives for medicine and public health. Improved wellbeing at lower costs [version 1; referees: 2 approved]. *F1000Research*.2016;5(167) : 10.5256/f1000research.8450.r12424 10.5256/f1000research.8450.r12424 PMC483798427134730

[2] LewisS: Exploring the Biological Meaning of Disease and Health. *last accessed 24th June 2016*.2003; Reference source

[3] SturmbergJPMartinCM: Diagnosis - the limiting focus of taxonomy. *J Eval Clin Pract*.2014; 10.1111/jep.12113 10.1111/jep.12113 24548570

[4] RosenbergCE: The tyranny of diagnosis: specific entities and individual experience. *Milbank Q*.2002;80(2) :237-60 1210187210.1111/1468-0009.t01-1-00003PMC2690110

[5] KonitzerMFinkWLipatovVKamenskiGKnippeT: Coping with Complexity and Uncertainty: Insights from Studying Epidemiology in Family Medicine. *In: Sturmberg JP (ed) The Value of Systems and Complexity Sciences for Healthcare, Springer International Publishing, Switzerland*.2016; 10.1007/978-3-319-26221-5_5 10.1007/978-3-319-26221-5_5 Reference source
